# Dose-Dependent Induction of an Idiotypic Cascade by Anti-Glycosaminoglycan Monoclonal Antibody in apoE^−/−^ Mice: Association with Atheroprotection

**DOI:** 10.3389/fimmu.2017.00232

**Published:** 2017-03-03

**Authors:** Roger Sarduy, Victor Brito, Adriana Castillo, Yosdel Soto, Tania Griñán, Sylvie Marleau, Ana María Vázquez

**Affiliations:** ^1^Division of Immunobiology, Center of Molecular Immunology, Havana, Cuba; ^2^Faculté de Pharmacie, Université de Montréal, Montréal, QC, Canada; ^3^Innovation Managing Direction, Center of Molecular Immunology, Havana, Cuba

**Keywords:** atherosclerosis, glycosaminoglycans, monoclonal antibody, idiotypic cascade, atheroprotection, antibodies

## Abstract

Atherosclerosis, the underlying pathology of most cardiovascular diseases, is triggered by the retention of apolipoprotein B (apoB)-containing lipoproteins in the arterial wall through electrostatic interactions with glycosaminoglycan (GAG) side chains of proteoglycans. Previously, we reported the antiatherogenic properties of the chimeric monoclonal antibody (mAb) chP3R99-LALA, which binds sulfated GAGs, inhibits low-density lipoprotein (LDL)–chondroitin sulfate (CS) association, and abrogates LDL oxidation and foam cell formation. In preventive and therapeutic settings, apoE-deficient (apoE^−/−^) mice immunized with 50 μg of this mAb showed reduced atherosclerotic lesions related with the induction of autologous anti-GAG antibodies. Knowing that age and sex are major non-modifiable risk factors in the development of atherosclerosis, the present study aimed to assess the influence of these variables on the capacity of chP3R99-LALA mAb to generate an anti-CS antibody response. Also, we aimed at defining the impact of the dose of chP3R99-LALA on the anti-CS antibody induction and the atheroprotective effect of this mAb in apoE^−/−^ mice. Neither age nor sex had an impact in the IgG anti-CS antibody response induced by s.c. immunization with this mAb. Moreover, chP3R99-LALA mAb reduced atherosclerotic lesions to a similar extent in both young male and female apoE^−/−^ mice fed a hypercholesterolemic diet and, in middle-aged female apoE^−/−^ mice, with spontaneous lesions. On the other hand, increasing the dose of chP3R99-LALA (200 vs. 50 μg) elicited an anti-idiotype antibody cascade characterized by higher levels of anti-idiotype (Ab2), anti-anti-idiotype (Ab3), and anti-CS antibody responses. Moreover, this dose increment resulted in a striking reduction of aortic atherosclerotic lesions in immunized mice.

## Introduction

Cardiovascular diseases (CVDs) still prevail as the leading cause of death worldwide (1). Furthermore, it was estimated that, by 2025, more than five million men and nearly three million women might die prematurely due to CVD (2). Atherosclerosis, the underlying pathology in most CVD, is initiated by the subendothelial retention of apoB-containing lipoproteins (3–5). This retention occurs by electrostatic interactions between glycosaminoglycan (GAG) chains and basic residues present in apoB-containing lipoproteins (6, 7). The trapped lipoproteins undergo oxidative and enzymatic modifications that trigger a maladaptive, non-resolving inflammatory process (7–13). In humans, epidemiological studies have demonstrated that different risk factors, including hypercholesterolemia, hypertension, diabetes, smoking, age, and gender influence the development and progression of atherosclerosis (14–17). Paralleling, what happens in humans, apoE-deficient (apoE^−/−^) mice, one of the most widely used murine model of atherosclerosis (18), exhibit accelerated atherosclerosis progression when fed a high-fat high-cholesterol (HFHC) diet (19, 20). Also, aging increases atherosclerosis in both male and female mice, although the influence of gender in the extent of the disease remains controversial (21–24).

Previously, we characterized the antiatherogenic properties of the chimeric murine/human monoclonal antibody (mAb) chP3R99 and its variant with impaired Fcγ receptor and complement binding (chP3R99-LALA), which bind sulfated GAGs, inhibit low-density lipoprotein (LDL)–chondroitin sulfate (CS) interaction, abrogate LDL oxidation *in vitro* and *in vivo* and, preferentially, accumulate in arterial lesions (25, 26). In preventive and therapeutic settings, male young adult New Zealand White rabbits and apoE^−/−^ mice immunized with this mAb showed reduced atherosclerotic lesions, associated with the induction of autologous anti-GAG antibodies generated due to the activation of an anti-idiotype antibody cascade (Ab2, Ab3, etc.) (25–27).

In an effort to expand our previous knowledge on the capacity of this mAb to activate such idiotypic network in apoE^−/−^ mice, now we evaluated (i) the influence of age and gender in the induction of autologous anti-CS antibodies by chP3R99-LALA immunization, (ii) the effect of different doses of the mAb in the induction of the anti-idiotype antibody cascade, and (iii) the effect of the increase of chP3R99-LALA mAb dose in aortic atherosclerosis lesions of apoE^−/−^ mice fed a HFHC diet.

## Materials and Methods

### Monoclonal Antibodies and Reagents

chP3R99-LALA (25), hR3 (28), and ch1E10 (29) IgG1 mAb were generated at the Center of Molecular Immunology (Havana, Cuba). ch1E10 is an anti-idiotype mAb that specifically reacts with chP3R99-LALA mAb variable regions. hR3 is an anti-human epidermal growth factor receptor antibody used as isotype-matched control antibody. The mAbs were purified from culture supernatants by protein-A affinity chromatography (Pharmacia, Uppsala, Sweden) and analyzed by sodium dodecyl sulfate polyacrylamide gel electrophoresis (SDS-PAGE) under reducing conditions. The specificity of the purified antibodies was confirmed by enzyme-linked immunoadsorbent assay (ELISA) and protein concentration was estimated by optical density (OD) at 280 nm. CS from bovine trachea was obtained from Sigma-Aldrich (St. Louis, MO, USA).

### Animals

Male and female apoE^−/−^ mice were housed under standard conditions (25°C, 60 ± 10% humidity) and exposed to 12 h light/dark cycle, with food and water *ad libitum*. The studies were approved by the Institutional Animal Ethic Committee of the Center of Molecular Immunology, in accordance with its animal care and use guidelines.

In a first series of experiments, adolescent (6-week olds) (30), young adult (16-week olds), middle-aged/old (56- to 76-week olds) male, and middle-aged (35-week olds) female apoE^−/−^ mice (31) fed a chow diet received four s.c. injections of 50 μg of chP3R99-LALA mAb at weekly intervals, followed by two additional biweekly injections. Blood samples were drawn and sera were obtained before treatment and 1 week after the fourth, fifth, and sixth injection of the mAb.

In a second experimental design, age-matched male and female apoE^−/−^ mice were fed with a HFHC diet containing 20% (wt/wt) lard and 1% (wt/wt) cholesterol (Sigma-Aldrich) from 6 weeks of age. Mice were treated with s.c. injections of either 50 μg of chP3R99-LALA or hR3 mAb at 12, 13, 14, 15, 17, and 19 weeks of age. In addition, middle-aged female apoE^−/−^ mice fed a chow diet underwent the same immunization protocol, as shown in Figure S2A in Supplementary Material.

In a third series of experiments, 6-week-old male apoE^−/−^ mice fed a chow diet were treated with four weekly s.c. injections of 50 or 200 μg of chP3R99-LALA mAb. Blood samples were obtained at the beginning of the study and a week after the administration of the third and fourth injection.

In a fourth experimental protocol, male apoE^−/−^ mice fed with the HFHC diet were treated with s.c. injections of 50 or 200 μg of chP3R99-LALA mAb or with 200 μg of isotype-matched mAb hR3, at 12, 13, 14, 15, 17, and 19 weeks of age.

Also, 1 week after the last immunization, the mice were anesthetized with ketamine hydrochloride (5 mg/kg i.m.) and euthanized with an overdose of sodium pentobarbital (90 mg/kg, i.v.) (Abbott Laboratories, Mexico City, Mexico). In the second and fourth experimental protocol, the vascular system was perfused with 0.9% NaCl solution at 4°C and then, whole aortas were isolated for morphometric analysis.

### Morphometric Analysis of Aortic Lesions

Aortas were opened longitudinally from the heart to the iliac arteries, and the lesions were stained with Oil-Red-O, as described previously (32). En face stained-aortas were digitized by light microscopy and analyzed using Adobe Photoshop CS3 software (Adobe Systems Incorporated, San José, CA, USA) and expressed as the percentage of the total aortic surface area covered by lesions.

### Reactivity of Sera against Chimeric Antibodies

Reactivity of immune sera against chimeric antibodies was determined by solid-phase ELISA as previously described (27). Briefly, 96-well Maxisorp polystyrene plates (Nunc, Roskilde, Denmark) were coated with 10 μg/mL of chP3R99-LALA, ch1E10, or hR3 mAb and incubated overnight at 4°C. Diluted sera (1/1,000) were added and the plates were incubated for 1 h at 37°C. Peroxidase-conjugated goat anti-mouse IgG secondary antibody (Jackson ImmunoResearch Laboratories, West Grove, PA, USA) was used to determine the reactivity associated with IgG fraction using 0.4 mg/mL of ortho-phenylenediamine in citrate/phosphate buffer, pH 5 containing 0.2% of H_2_O_2._ Sera reactivity is expressed as OD values at 490 nm.

### Reactivity of Sera against CS

The presence of antibodies against CS was assessed by solid-phase ELISA, as previously described (27). Maxisorp polystyrene ELISA plates (Nunc) were coated with 10 μg/mL of CS and incubated overnight at 4°C. Mice sera (1/400) was added to the plates and incubated for 1 h at room temperature. Peroxidase-conjugated goat anti-mouse IgG secondary antibody (Jackson ImmunoResearch Laboratories) was added and the plates incubated for 1 h at room temperature. Colorimetric reaction was developed using 0.4 mg/mL of ortho-phenylenediamine in citrate/phosphate buffer, pH 5 containing 0.2% of H_2_O_2_. Sera reactivity is expressed as OD values at 490 nm.

The pattern of IgG subclasses developed in immunized mice was determined using biotinylated goat anti-mouse IgG1, IgG2a, IgG2b, or IgG3 (BD Pharmingen, San Diego, CA, USA), followed by incubation with streptavidin-conjugated to alkaline phosphatase (Jackson ImmunoResearch Laboratories) for 30 min at 37°C. The reaction was developed using 1 mg/mL of para-nitrophenyl phosphate in diethanolamine buffer solution pH 9.

Antibody response induction was considered positive when hyperimmune serum OD was ≥0.2 and at least twofold the preimmune serum OD value. Assays were performed in triplicate for each sample and the coefficient of variation was <15% for all values.

### Statistical Analysis

All data are represented as mean ± SEM. Statistical analysis was performed with the GraphPad Prism version 6.0 software (GraphPad Software Inc., San Diego, CA, USA). Comparisons between independent groups were performed using one-way ANOVA followed by Tukey *post hoc* test or Student’s *t*-test as appropriate.

## Results

### chP3R99-LALA mAb Induced Anti-CS Autologous Antibody Response Independently of Age and Gender in ApoE^−/−^ Mice Fed a Chow Diet

**Figure 1:**
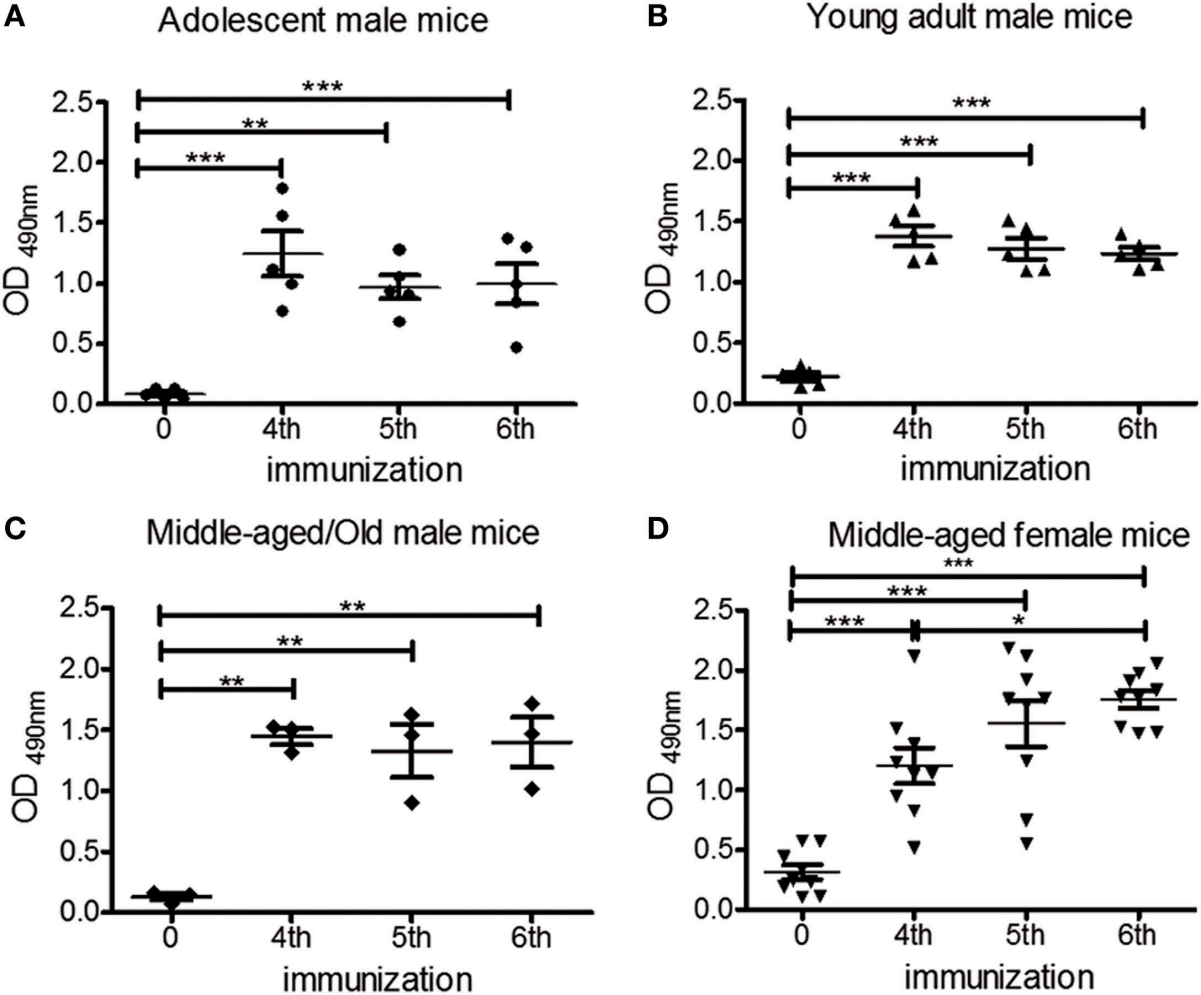
**Effect of age and gender on the induction of anti-chondroitin sulfate (CS) antibody response in apoE^−/−^ mice immunized with chP3R99-LALA monoclonal antibody (mAb)**. Mice fed a chow diet received four s.c. injections of 50 μg of chP3R99-LALA mAb at weekly intervals and two additional immunizations biweekly. Sera were obtained prior to the first antibody injection and after the fourth, fifth and sixth immunization of **(A)** adolescent (6-week olds, *n* = 5), **(B)** young adult (16-week olds, *n* = 5), **(C)** middle-aged/old (56- to 76-week olds, *n* = 3) male, and **(D)** middle-aged (35-week olds, *n* = 9) female apoE^−/−^ mice. To measure the kinetics of the response against CS, sera from immunized mice (diluted 1:400) were added to enzyme-linked immunoadsorbent assay plates coated with 10 μg/mL of CS and the reaction was developed with peroxidase-conjugated goat anti-mouse IgG. The levels of anti-CS antibodies are expressed as optical density (OD) values. Results are mean ± SEM. *P < 0.05, **P < 0.01, ***P < 0.001, one-way ANOVA followed by Tukey *post hoc* test.

To assess the effect of aging in the capacity of chP3R99-LALA mAb to induce an anti-CS antibody response, adolescent, young adult, and middle-aged/old, apoE^−/−^ male mice received six s.c. injections of 50 μg of the mAb. The presence of anti-CS autologous antibodies was measured in pre- and hyperimmune serum samples through their binding to CS-coated ELISA microplates. IgG anti-CS levels were significantly higher in hyperimmune sera from all mice groups in comparison with those detected in the preimmune sera (Figures 1A–C, *P* < 0.0001). To evaluate the impact of gender in the induction of anti-CS antibody response by the administration of chP3R99-LALA mAb, middle-aged female apoE^−/−^ mice were immunized with the same immunization schedule. Hyperimmune sera also displayed a stronger reactivity against CS compared with preimmune sera, as observed for males (Figure 1D, *P* < 0.0001). These autologous antibody responses reached a plateau in all groups after the mice received the fourth or fifth injection of chP3R99-LALA mAb (Figures 1A–D). No differences were found in anti-CS antibody levels irrespective of age and gender after the sixth immunization (Figure S1 in Supplementary Material).

### chP3R99-LALA mAb Had a Similar Antiatherosclerotic Effect in ApoE^−/−^ Mice of Both Genders

**Figure 2:**
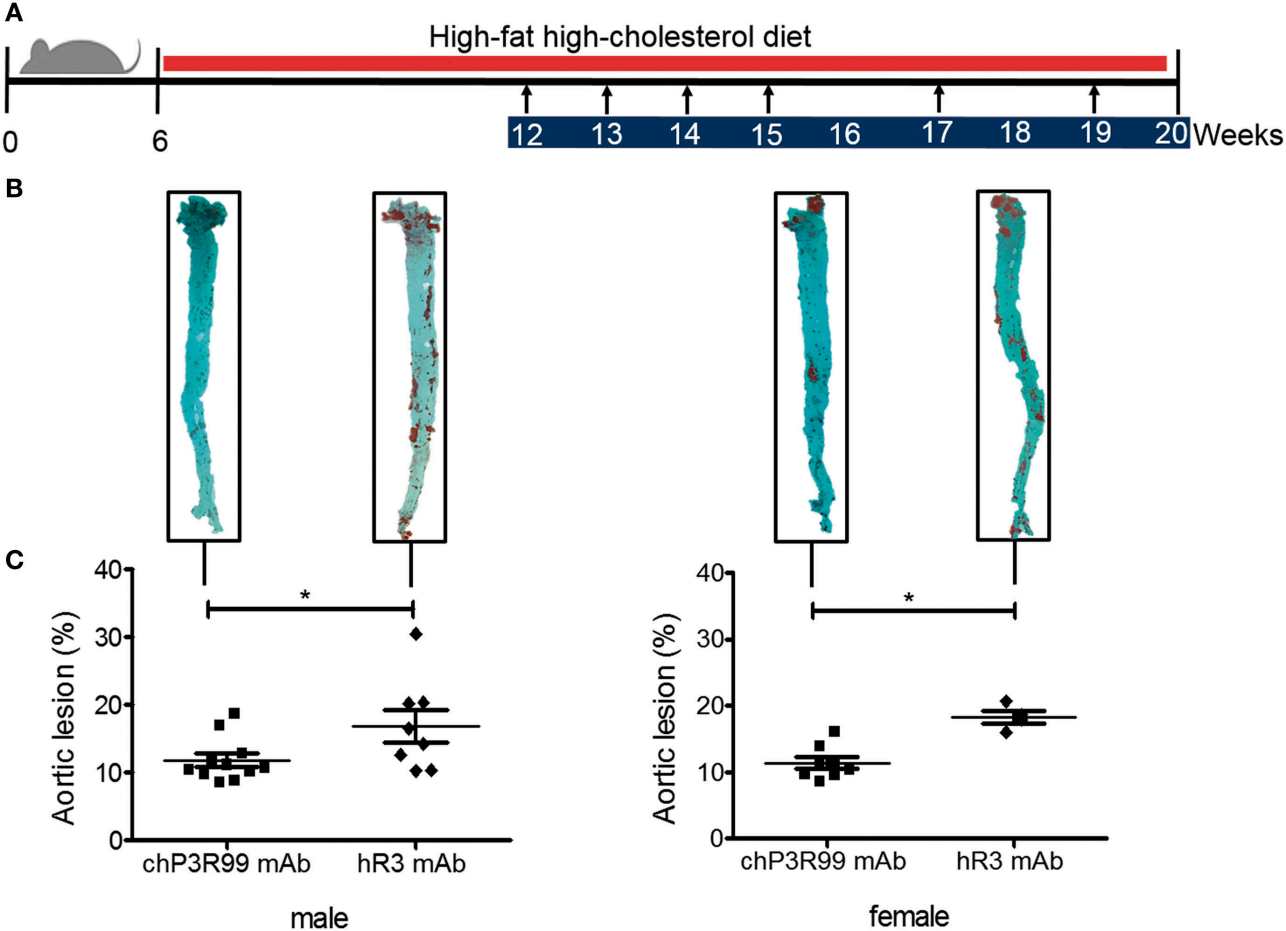
**Effect of immunization with chP3R99-LALA monoclonal antibody (mAb) on atherosclerosis development in apoE^−/−^ mice of both genders**. **(A)** Experimental design: arrows represent immunization time points with 50 μg of chP3R99-LALA mAb or the isotype-matched control hR3 mAb. Blue and red bars indicate treatment and high-fat high-cholesterol diet period, respectively. **(B)** Representative en face Oil-Red-O stained aortas from each experimental group. **(C)** Mean percentage of aortic lesion areas in male and female apoE^−/−^ mice treated with chP3R99-LALA (*n* = 11 or 8) or hR3 (*n* = 8 or 4). Results are mean ± SEM. *P < 0.05, Student’s *t*-test.

Next, we evaluated whether similar induced anti-CS antibody responses between male and female apoE^−/−^ mice treated with chP3R99-LALA mAb would confer similar atheroprotection. The antiatherogenic effect of chP3R99-LALA mAb was assessed in apoE^−/−^ mice of both gender fed a HFHC diet from 6 weeks of age, and injected s.c. with six doses (50 μg/dose) of chP3R99-LALA mAb or isotype control mAb hR3, starting at 12-week olds according to the schedule shown in Figure 2A. At the end of the experiment, lipid accumulation in whole aortas was detected by en face staining with Oil-Red-O. Immunization with chP3R99-LALA mAb similarly reduced the mean aortic lesion area by 31% (*P* < 0.05) and 38% (*P* < 0.05) in male and female mice, respectively, compared with their respective isotype-matched control-treated group, even though the atherogenic diet was maintained during all the immunization protocol (Figures 2B,C). Interestingly, a similar decrease in the mean percent aortic lesion area (31%, *P* < 0.05) was observed in 35-week-old female apoE^−/−^ mice with spontaneous development of atherosclerotic lesions, immunized with six doses of chP3R99-LALA mAb (50 μg/dose), in comparison with isotype-matched control treatment (Figure S2 in Supplementary Material).

### The Anti-CS Antibody Response Induced in ApoE^−/−^ Mice Was Dependent on the Dose of chP3R99-LALA mAb Administered

**Figure 3:**
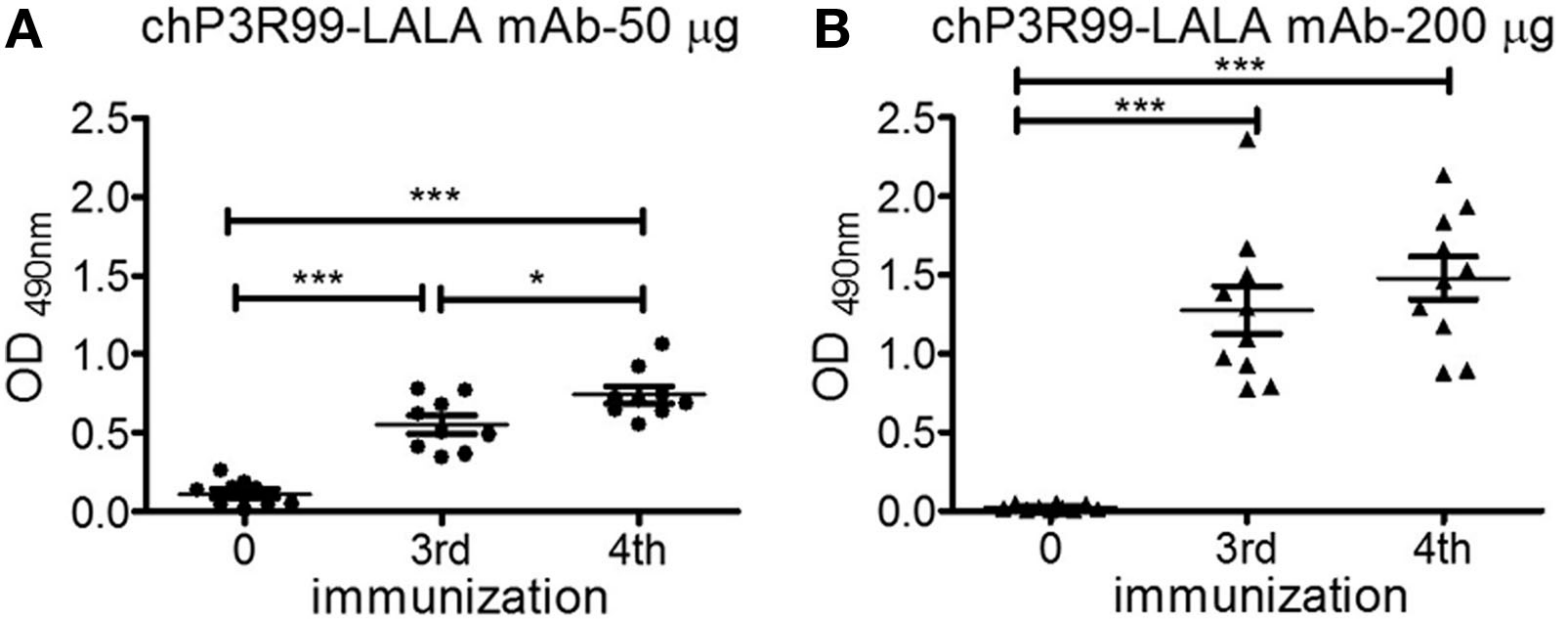
**Antibody response to chondroitin sulfate (CS) in the sera of apoE^−/−^ mice immunized with different doses of chP3R99-LALA monoclonal antibody (mAb)**. A 6-week-old mice fed with chow diet received four weekly s.c. injections of **(A)** 50 μg (*n* = 9) or **(B)** 200 μg (*n* = 10) of chP3R99-LALA mAb. Sera obtained before starting immunizations and 1 week after the third and fourth antibody injection (diluted 1:400) were added to enzyme-linked immunoadsorbent assay plates coated with 10 μg/mL of CS and the reaction was developed with peroxidase-conjugated goat anti-mouse IgG. The levels of IgG are expressed as OD values. Results are mean ± SEM. *P < 0.05, ***P < 0.001, one-way ANOVA followed by Tukey *post hoc* test.

To evaluate the effect of increasing dose of chP3R99-LALA mAb on the anti-CS antibody response, 6-week-old male apoE^−/−^ mice fed with chow diet received four injections of 50 or 200 μg of the antibody. IgG anti-CS response augmented with the number of administered injections in the 50 μg-treated group (Figure 3A, *P* < 0.0001), whereas the administration of 200 μg of chP3R99-LALA increased the antibody response in hyperimmune sera (*P* < 0.0001), reaching a plateau after mice received three injections (Figure 3B). The immunization with 200 μg of chP3R99-LALA induced higher anti-CS antibody response than 50 μg when this response was measured in mice sera 7 days after the third (1.28 ± 0.15 vs. 0.55 ± 0.06, *P* < 0.0001) and the fourth (1.48 ± 0.13 vs. 0.74 ± 0.05, *P* < 0.0001) administration of the mAb. The anti-CS response induced exclusively antibodies of IgG1 subclass (data not shown).

### The Anti-Idiotype (Ab2) and Anti-Anti-Idiotype Antibody (Ab3) Responses Induced in ApoE^−/−^ Mice Were Dependent on the Dose of chP3R99-LALA mAb Administered

**Figure 4:**
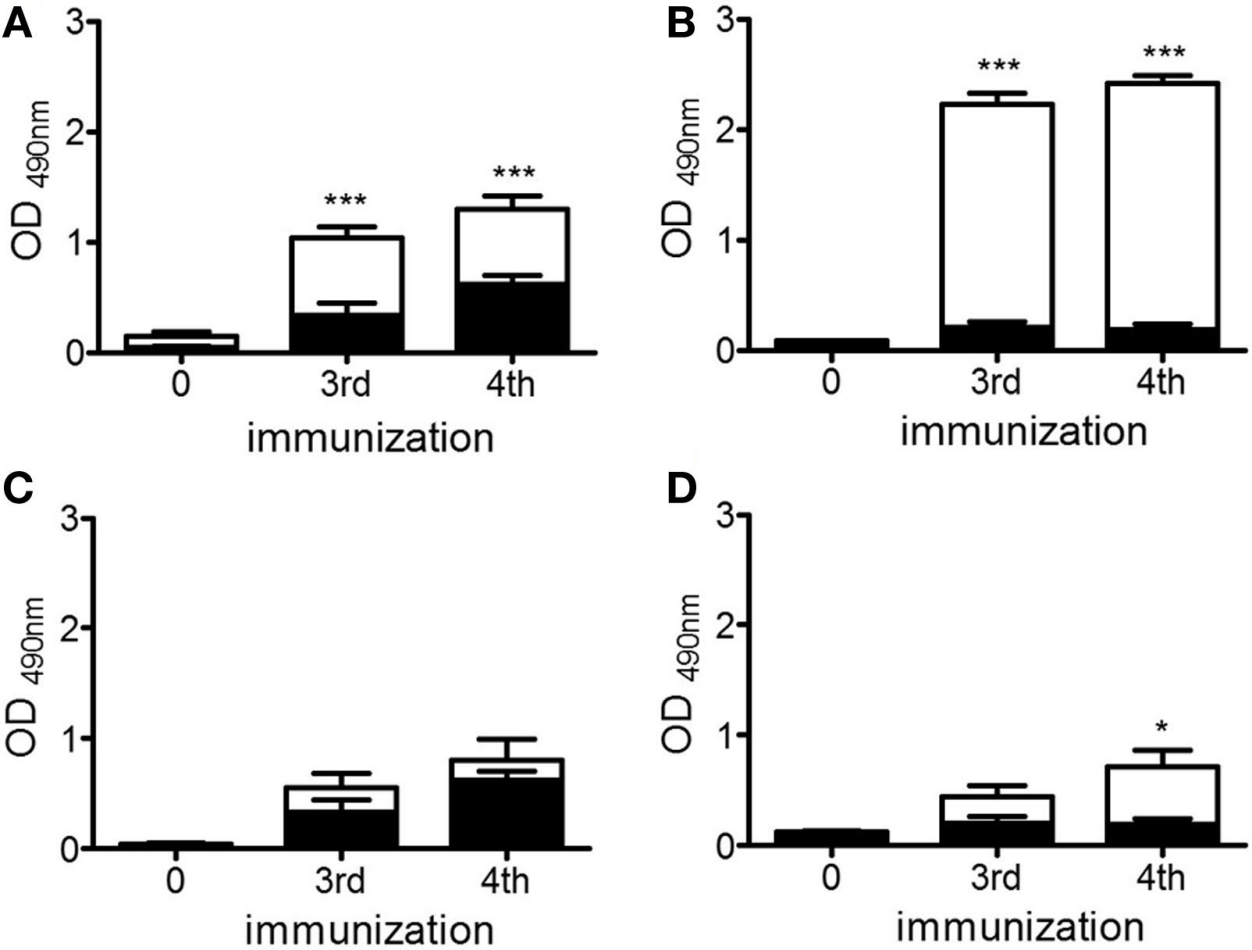
**Anti-idiotype (A,B) and anti-anti-idiotype (C,D) antibody responses induced in apoE^−/−^ mice by immunization with doses of 50 μg (A,C) and 200 μg (B,D) of chP3R99-LALA monoclonal antibody (mAb)**. The anti-idiotype **(A,B)** and anti-anti-idiotype **(C,D)** antibody responses were measured by enzyme-linked immunoadsorbent assay, adding mice sera (diluted 1:1,000) to chP3R99-LALA mAb- or ch1E10 Ab2 mAb-coated plates, respectively (white bars). hR3 mAb-coated plates were used to measure the anti-isotype response (black bars). The reaction was developed with peroxidase-conjugated goat anti-mouse IgG. Sera were obtained before the first antibody injection and 1 week after the third and fourth immunization. The serological antibody responses are expressed as OD values. Results are mean ± SEM. *P < 0.05, ***P < 0.001, Student’s *t*-test when the levels of anti-chP3R99-LALA or anti-ch1E10 mAb serological antibody responses were compared with those against hR3 mAb.

Knowing that the immunization with chP3R99-LALA mAb (Ab1) is able to induce an anti-idiotype antibody cascade in apoE^−/−^ mice (27), we evaluated whether the increase in the anti-CS antibody response detected in apoE^−/−^ mice treated with 200 μg of the mAb was associated with an increase of the anti-idiotypic (Ab2) and anti-anti-idiotypic (Ab3) antibody responses. To evaluate the dose-dependence of the Ab2 response, serological IgG reactivities against chP3R99-LALA whole molecule and the isotype-matched-control mAb (hR3) were determined by ELISA. The injection of 50 μg (Figure 4A) and 200 μg (Figure 4B) generated a higher antibody immune response against chP3R99-LALA molecule than against the isotype-matched antibody control (*P* < 0.0001), showing the immunodominance of chP3R99-LALA idiotype. However, four administrations of 200 μg of the mAb induced not only a higher level of anti-chP3R99 IgG antibodies than 50 μg (2.41 ± 0.07 vs. 1.29 ± 0.12, *P* < 0.0001) but also a lower response against the isotype (0.20 ± 0.05 vs. 0.62 ± 0.08, *P* < 0.001).

In addition, we determined the presence of Ab3 antibodies in the sera of chP3R99-LALA-treated mice, measuring by ELISA their reactivity against an Ab2 mAb that binds chP3R99-LALA-idiotype, named ch1E10. Although hyperimmune sera from apoE^−/−^ mice immunized with either 50 or 200 μg of chP3R99-LALA mAb reacted with ch1E10 mAb, only those sera from mice treated with four injections of the higher dose showed a significant specific binding to the Ab2 mAb in comparison with the reactivity against the isotype-matched antibody control hR3 (Figures 4C,D, *P* < 0.05).

### The Antiatherogenic Effect Generated by chP3R99-LALA mAb Immunization in ApoE^−/−^ Mice Was Dependent on the Dose

**Figure 5:**
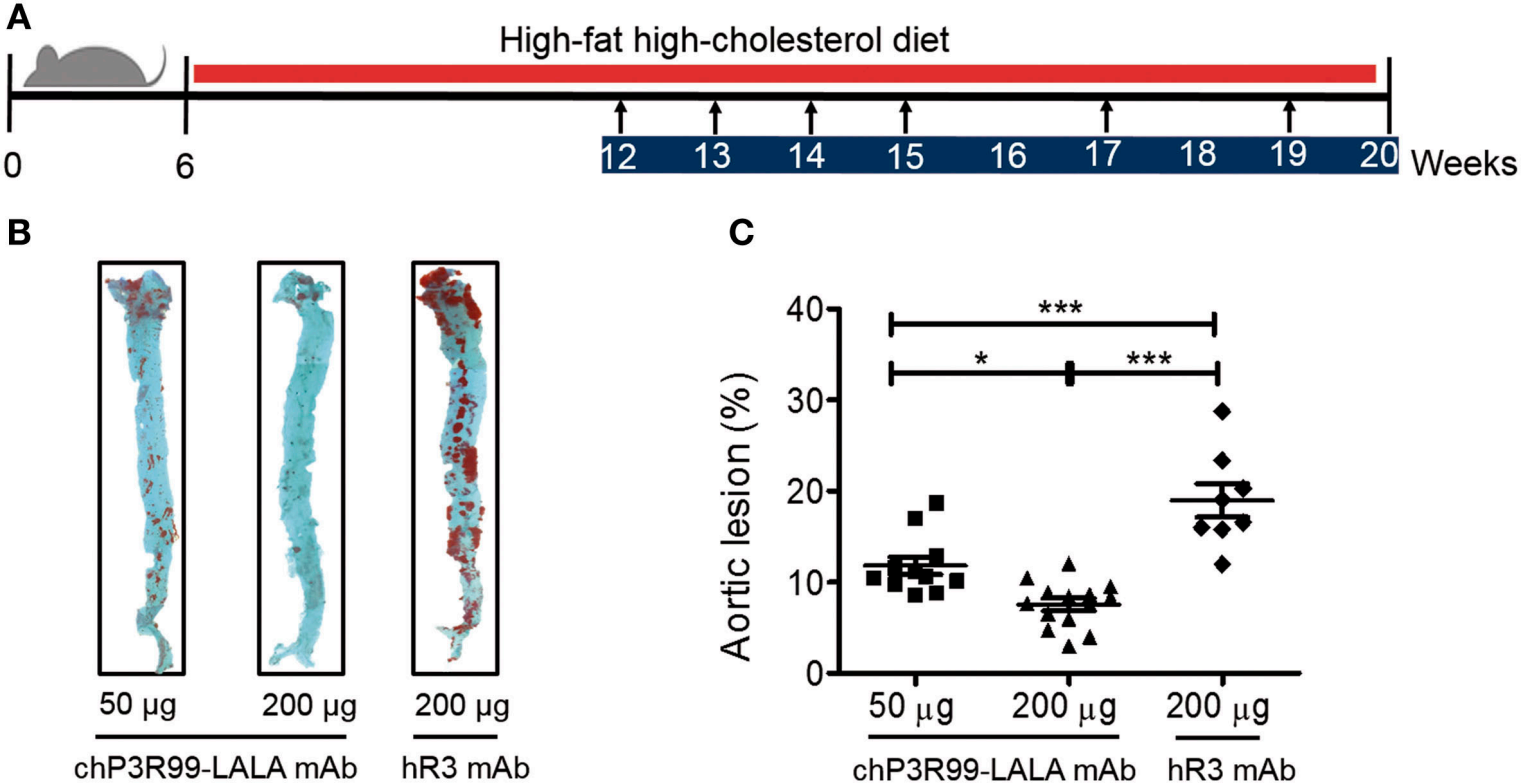
**Antiatherosclerotic effect induced by chP3R99-LALA monoclonal antibody (mAb) immunization at different doses in apoE^−/−^ mice**. **(A)** Experimental design: arrows represent immunization points according to the experimental procedure with 50 μg of chP3R99-LALA mAb (*n* = 11), 200 μg of chP3R99-LALA mAb (*n* = 14), or 200 μg of hR3 mAb (*n* = 8). **(B)** Representative en face Oil-Red-O-stained aortas from the different groups. **(C)** Mean percentage of aortic lesion area in apoE^−/−^ mice treated with 50 or 200 μg of chP3R99-LALA or hR3. Results are mean ± SEM. *P < 0.05, ***P < 0.001, one-way ANOVA followed by Tukey *post hoc* test.

Finally, we tested the effect of different doses of chP3R99-LALA mAb on the atherosclerotic lesion progression in apoE^−/−^ male mice fed HFHC diet from 6 to 20 weeks of age and treated with six injections of 50 or 200 μg of the antibody, starting the immunizations when animals were 12 weeks of age (Figure 5A). En face analysis of whole aortas showed that mean aortic lesion areas of 50 and 200 μg chP3R99-LALA-treated mice were reduced by 40% (*P* < 0.0001) and 60% (*P* < 0.0001) compared with the isotype-matched control mAb-treated group, respectively (Figures 5B,C).

## Discussion

Aging is one of the most important non-modifiable risk factor for the development of atherosclerosis in human and mice (15, 33). On the other hand, aged humans and mice display impaired T and B cell responses (24–31). In particular, antibody responses could be reduced in magnitude and the antibodies could be less protective compared with those induced in young adults (34–40). Therefore, any therapeutic approach directed to efficiently activate immune responses in aging diseases, such as atherosclerosis, must take into account this fact.

In addition to the known induction of autologous anti-GAG antibodies in adolescent and young adult apoE^−/−^ male mice immunized with chP3R99-LALA mAb (27, 41), we extend our previous findings by showing that in middle-aged and old apoE^−/−^ mice, immunization with chP3R99-LALA mAb induced anti-CS antibody responses of similar levels and kinetics as in young animals, irrespective of the gender. Indeed, a similar reduction in the percentage of aortic lesion area in 12-week-old male and female apoE^−/−^ mice fed a HFHC diet, when compared to their respective isotype-matched control antibody-treated groups, showed the capacity of chP3R99-LALA mAb to induce atheroprotection in both genders. In addition, the mean aortic area with spontaneous lesions was decreased in 35-week-old female apoE^−/−^ mice immunized with chP3R99-LALA compared to control treatment, in a similar fashion to young male and female apoE^−/−^ mice. This observation is in line with our findings of the induction of the anti-CS response and may suggest that the antiatherosclerotic capacity of chP3R99-LALA mAb is not influenced by age, at least across the age range evaluated.

In previous studies (27, 41), anti-GAG antibody response and antiatherogenic effect of 50 μg of chP3R99-LALA was tested. To address dose-dependency, we determined the anti-CS antibody response induced by a fourfold higher dose of chP3R99-LALA mAb. The results showed that the response plateaued earlier and the levels of serological anti-CS antibodies were significantly higher than those induced with 50 μg of the mAb. Improvement of anti-CS antibody response with 200 μg of chP3R99-LALA was associated with the generation of higher levels of Ab2 and Ab3 responses in apoE^−/−^ mice treated with this dose. A surprising result was that not only a higher antibody response against chP3R99-LALA idiotype was observed with 200 μg but also a significantly lower one was found against the isotype in comparison with 50 μg-immunized animals. One explanation for this finding could be that, although the human constant region of this chimeric antibody constitutes 70% of the immunoglobulin molecule, the murine idiotype of this mAb is more immunogenic and thus is immunodominant in the induction of the humoral response against chP3R99-LALA in mice (27). Accordingly, when the immune system of apoE^−/−^ mice was exposed to a higher dose of chP3R99-LALA molecules, an increased number of B-lymphocytes may be triggered to produce anti-idiotype antibodies and, in turn, less would produce antibodies reacting with the isotype. Interestingly, the IgG antibody response induced by chP3R99-LALA in immunized mice was restricted to the IgG1 subclass, which is associated with a Th2-like, non-inflammatory humoral response (42).

The demonstration of anti-GAG antibodies induced in chP3R99-LALA-immunized apoE^−/−^ mice was related to the activation of an anti-idiotype antibody cascade that generates Ab3 antibodies reacting with both CS and ch1E10 Ab2 mAb idiotype was reported by us previously (27). Along this line, now we show that, in accordance with the higher presence of autologous Ab2 antibodies in the sera of mice immunized with 200 μg of chP3R99-LALA mAb, a higher level of a fraction of Ab3 antibodies capable to react with ch1E10 Ab2 mAb was detected in the sera of these mice.

Finally, we assessed the impact of increasing the dose of chP3R99-LALA on the progression of atherosclerosis. Our results showed that the higher dose of the mAb also reduced more efficiently the percentage of aortic lesions produced by a HFHC diet in apoE^−/−^. Although a direct contribution of chP3R99-LALA mAb *per se* in the antiatherogenic effect might be possible due to its demonstrated preferential accumulation in atherosclerotic lesions (25, 27), the present results reinforce our previous data (25, 27, 41) showing an association between atheroprotection and the autologous anti-GAG antibody response induced by the immunization with the mAb.

Future kinetic studies should determine the accumulation in the arterial wall of chP3R99-LALA mAb and the autologous anti-GAG antibodies induced by its s.c. administration. In addition, experiments will be designed to define the minimum number of chP3R99-LALA injections needed to reach the higher atheroprotection and how long lasting will be this effect in the treated mice.

## Author Contributions

RS participated in the design of the study, carried out the experiments, collected and interpreted data, and drafted the manuscript. AC and TG performed experiment and analyzed data. VB, YS, and SM analyzed and interpreted data and critical reviewed the manuscript. AV conceived and designed the study, analyzed and interpreted the data, and wrote the paper. All the authors read and approved the final manuscript.

## Conflict of Interest Statement

AV, VB, and YS are inventors of patents related with P3 monoclonal antibody and its anti-idiotype and related with antibodies that react with sulfatides and sulfated PGs; however, they have signed the assignment of their rights to the assignee Center of Molecular Immunology. The other authors have no conflicts to report.
