# Supplementary material for: Dose-Dependent Induction of an Idiotypic Cascade by Anti-Glycosaminoglycan Monoclonal Antibody in apoE−/− Mice: Association with Atheroprotection
Source: Front Immunol. 2017 Mar 3;8:232. doi: 10.3389/fimmu.2017.00232 (PMC5334371; doi:10.3389/fimmu.2017.00232)
Supplement: Supplementary file 1 [file Presentation_1.PDF]

SUPPLEMENTARY MATERIAL

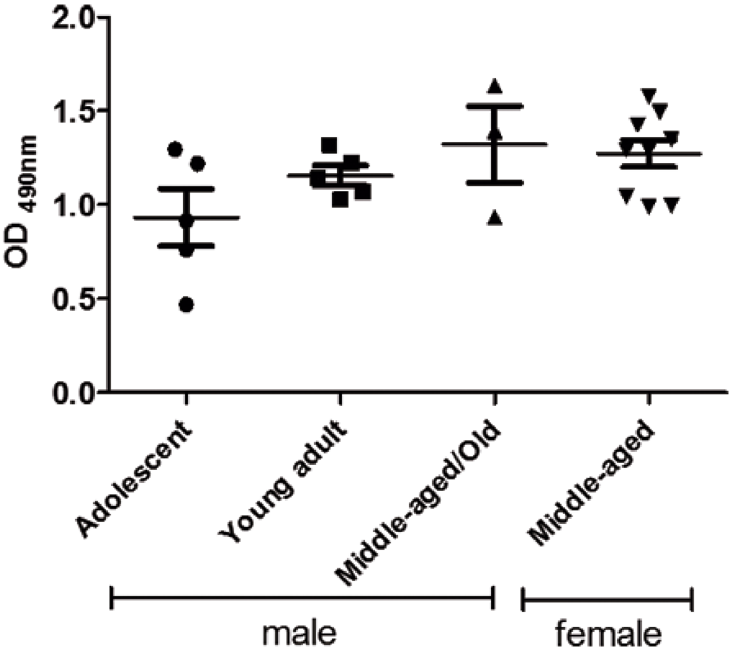

Supplementary Figure 1. **Comparison of the anti-CS antibody responses induced in apoE$^{-/-}$ mice with different age and gender immunized with six injections of chP3R99-LALA mAb.** Mice fed a chow diet received six s.c. injections of 50 µg of chP3R99-LALA mAb and the levels of anti-CS antibodies were measured by ELISA. Sera from immunized mice (diluted 1:400) were added to CS-coated ELISA plates (10 µg/mL) and the reaction was developed with peroxidase-conjugated goat anti-mouse IgG. The levels of serological IgG anti-CS antibodies are expressed as the optical density (OD) values from hyperimmune sera after the subtraction of OD values from preimmune sera. Results are mean ± SEM. $P > 0.05$, one-way ANOVA followed by Tukey post-hoc test.

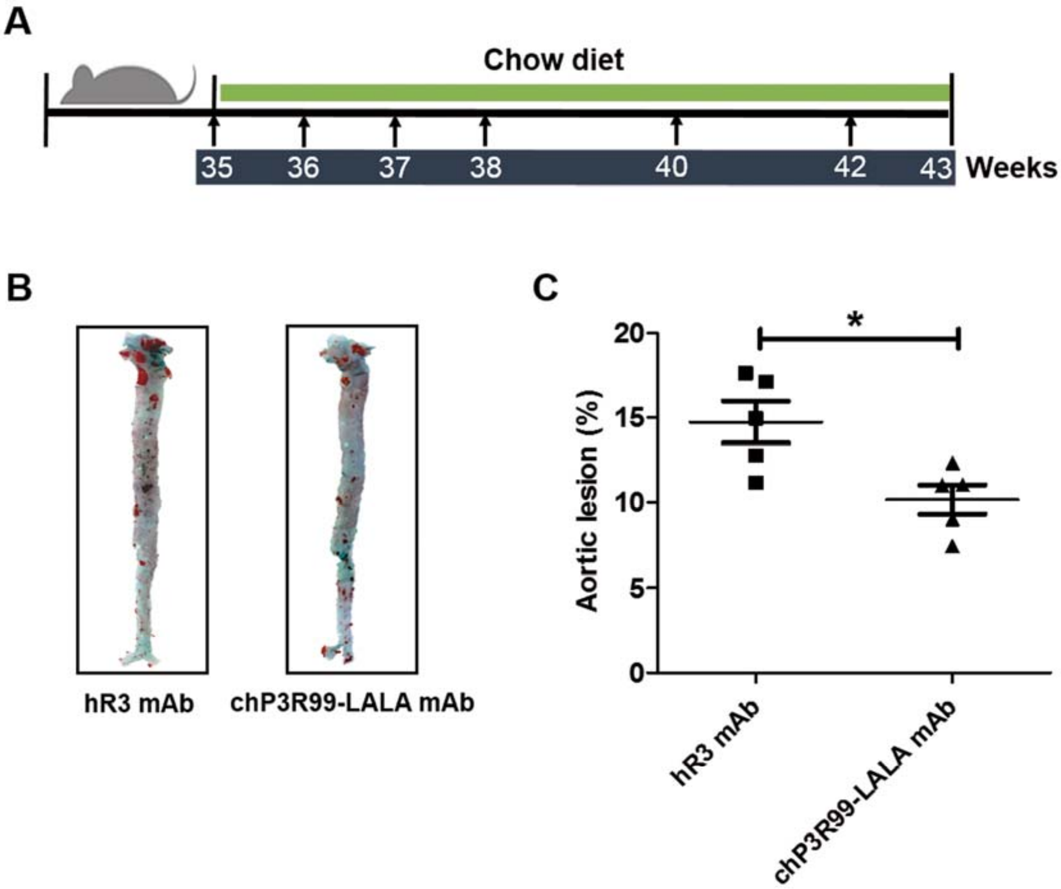

Supplementary Figure 2. **Effect of immunization with chP3R99-LALA mAb on spontaneous atherosclerosis development in middle-aged female apoE$^{-/-}$ mice. (A)** Experimental design: Arrows represent immunization time points with 50 µg of chP3R99-LALA mAb or the isotype-matched control hR3 mAb. Blue and green bars indicate treatment and chow diet period, respectively. **(B)** Representative en face Oil red-O stained aortas from each experimental group. **(C)** Mean percentage of aortic lesion areas in female apoE$^{-/-}$ mice treated with chP3R99-LALA (n = 5) or hR3 (n = 5). Results are mean ± SEM. *$P < 0.05$, Student's t-test.
